# Tunable High Refractive Index Polymer Hybrid and Polymer–Inorganic Nanocomposite Coatings

**DOI:** 10.1021/acsami.1c07372

**Published:** 2021-07-13

**Authors:** Angus W. Ritchie, Harrison J. Cox, Hassan I. Gonabadi, Steve J. Bull, Jas Pal S. Badyal

**Affiliations:** † Department of Chemistry, Durham University, Durham DH1 3LE, England, U.K.; ‡ School of Engineering, Newcastle University, Newcastle-upon-Tyne NE1 7RU, England, U.K.

**Keywords:** refractive index, hybrid polymer, polymer−inorganic, nanocomposite, atomized, plasma, coating

## Abstract

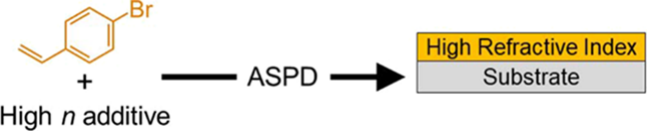

Atomized spray plasma deposition (ASPD) provides a single-step, low-temperature, and dry approach for the preparation of high refractive index hybrid polymer or polymer–inorganic nanocomposite coatings. Refractive indices as high as 1.936 at 635 nm wavelength have been obtained for ASPD 4-bromostyrene/toluene–TiO_2_ nanocomposite layers containing low titania loadings. Thin films with any desired refractive index up to 1.936 can be easily deposited onto a variety of substrates by varying the precursor mixture composition. ASPD overcomes disadvantages commonly associated with alternative fabrication methods for depositing high refractive index coatings (elevated temperatures, wet processes, UV curing steps, and much greater inorganic loadings).

## 1 Introduction

High refractive index materials attract a great deal of interest because of their wide range of technological applications, including optical lenses,^1^ waveguides,^2^ antireflective coatings,^3^ and as encapsulants for both light-emitting diodes (LEDs)^4^ and photovoltaic cells.^5^ Given that inorganic materials have an inherently high refractive index (*n*), one approach to achieve even higher refractive index coatings is through the use of pure inorganic thin films. Coatings of ZnO, ZrO_2_, and TiO_2_ are reported to have refractive indices of 1.87,^6^ 1.96,^7^ and 2.28,^8^ respectively. However, these coatings require multiple-step fabrication procedures and elevated temperatures and can be incompatible with common plastic substrates. Most common polymer materials have a refractive index between 1.3 and 1.7.^9^ More recently, high refractive index polymers (HRIPs) with refractive indices as high as 1.85 have been reported.^10−12^ These HRIPs are prepared by introducing atoms or substituents of high molar refraction into the polymer chain. Such substituents include aromatic rings,^13^ halogens (except for fluorine),^14^ sulfur,^15^ phosphorus,^16^ and silicon.^17^ Although these materials offer the advantages of being lightweight and easy to process, their preparation also requires lengthy and costly synthetic procedures.^18^

Hybrid organic–inorganic composite materials potentially offer high refractive indices and can be prepared using a variety of different methods. The most common is the sol–gel method in which a metal alkoxide precursor is mixed with an organic compound followed by heating during which hydrolysis and condensation of the metal alkoxide leads to the formation of metal oxide domains within an organic matrix coating (refractive index values, *n* = 1.7–1.9).^19−22^ Sol–gel coatings containing TiO_2_ are reported with refractive index values of *n* = 1.92–2.05, but the inorganic loading is high (49–90 wt %).^23−25^ Other types of high refractive index composite coatings contain inorganic materials such as PbS,^26^ Si,^27^ and V_2_O_5_;^28^ however, these require, for instance, the use of H_2_S gas^26^ or a complex milling process,^27^ and the coatings absorb strongly in the visible wavelength range.^28^ In all of these cases, the inorganic content also tends to be high (40–90 wt %) and the synthesis can be lengthy involving wet processing and elevated temperatures.

An alternative approach for fabricating organic–inorganic composite coatings is dispersing inorganic nanoparticles into an organic polymer prior to application onto substrates. A key requirement of this method is the use of nanoparticles (<25 nm in size, which is well below one tenth of the wavelength of visible light) in order to avoid poor optical transparency due to Rayleigh scattering.^29^ As with sol–gel coatings, high refractive indices in the range of 1.7–1.972 can be attained but again rely upon high levels of inorganic material loadings (45–97 wt %).^30−32^ Carbon black particles have been used at lower loadings (10 wt %, *n* = 1.833); however, the coating transparency can be comprised as a consequence of agglomeration.^33^ It is also possible to disperse inorganic nanoparticles into monomers prior to polymerization.^34,35^ Refractive indices as high as *n* = 1.972 are reported for this approach at 50 wt % loading of graphene oxide.^36^ As with the sol–gel coatings, these nanoparticle/monomer dispersions and nanoparticle/polymer dispersions all require high temperatures (120–300 °C) or additional UV curing steps. Therefore, a demand exists for ambient temperature, single-step fabrication methods for high refractive index coatings containing low levels of inorganic materials.

**Scheme 1:**
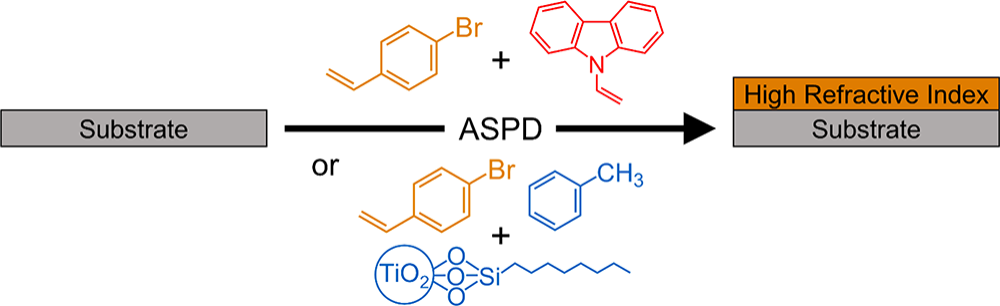
Atomized Spray Plasma Deposition of 4-Bromostyrene–9-vinylcarbazole Hybrid and 4-Bromostyrene/Toluene + TiO_2_ Nanocomposite High Refractive Index Layers

Atomized spray plasma deposition (ASPD) is a scalable, single-step, room temperature, and dry method for the preparation of functional coatings.^37−39^ This encompasses the nebulization of liquid or slurry droplets into a non-equilibrium electrical discharge. At low energy inputs, high levels of structural retention (functionality) can be attained. In this article, high refractive index polymer coatings are prepared by ASPD using a 4-bromostyrene precursor (*n* = 1.595^40^). The refractive index is further increased by mixing the 4-bromostyrene precursor for ASPD with a higher refractive index solid (9-vinylcarbazole, *n* = 1.683^41^) or functionalized titania nanoparticles (*n*_anatase_ = 2.45; *n*_rutile_ = 2.70^20^), leading to the deposition of high refractive index hybrid polymer or polymer–titania nanocomposite coatings, respectively, Scheme 1.

## 2 Experimental Section

### 2.1 Atomized Spray Plasma Deposition

**Figure 1:**
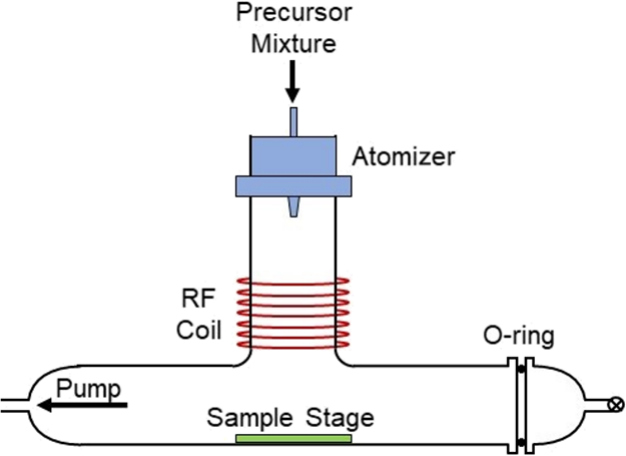
Atomized spray plasma deposition (ASPD) chamber.^37,43^

Precursor materials used were 4-bromostyrene (+95%, Apollo Scientific Ltd.), 9-vinylcarbazole (98%, Sigma-Aldrich Ltd.), and trimethoxyoctylsilane-functionalized titania nanoparticles (21 nm average particle size, Aeroxide T805, Evonik Industries AG). For the case of the 4-bromostyrene precursor mixed with trimethoxyoctylsilane-functionalized titania nanoparticles, 20 or 40% v/v of toluene (99.99 wt %, Fisher Scientific Ltd.) was added to improve dispersion.^42^ Liquid–solid slurry monomer–nanoparticle mixtures were sonicated for 60 min to fully disperse the nanoparticles (Clifton ultrasonic bath, Nickel-Electro Ltd.) and then loaded into a sealable glass delivery tube. This precursor slurry mixture was then degassed using multiple freeze–pump–thaw cycles. Substrates used for coating were silicon (100) wafers (0.014–0.024 Ω cm resistivity, Silicon Valley Microelectronics Inc.), quartz slides (20 mm × 10 mm × 1 mm, UQG Ltd.), and polyethylene terephthalate film (PET, capacitor grade, 0.10 mm thickness, Lawson Mardon Ltd.). These were cleaned by sonication in a 1:1 v/v propan-2-ol (+99.5 wt %, Fisher Scientific Ltd.)/cyclohexane (+99.5%, Fisher Scientific Ltd.) solvent mixture, followed by UV–ozone treatment (model UV.TC.EU.003, BioForce Nanosciences Inc.), and a final sonication step in the propan-2-ol/cyclohexane solvent mixture. After air drying, substrates were placed downstream in line-of-sight from the ASPD atomizer, Figure 1.

Atomized spray plasma deposition (ASPD) was carried out in an electrodeless, cylindrical, T-shape glass reactor (volume of 1195 cm^3^, base pressure of less than 3 × 10^–3^ mbar, and a leak rate better than 2 × 10^–9^ mol s^–1^) enclosed in a Faraday cage.^44^ The chamber was pumped by a 30 L min^–1^ two-stage rotary pump (model E2M2, Edwards Vacuum Ltd.) attached to a liquid nitrogen cold trap, and the system pressure was monitored by a thermocouple gauge. An L–C impedance matching network was used to minimize the standing wave ratio for power transmitted from a 13.56 MHz radio frequency (RF) power supply to a copper coil (4 mm diameter, 7 turns, spanning 5.5 cm). The copper coil was located 12.5 cm downstream from the atomizer nozzle (18 μm diameter median droplet size, model no. 8700-120, Sono-Tek Corp.^45,46^), which was driven by a broadband ultrasonic generator (120 kHz, model no. 06-05108, Sono-Tek Corp.). Prior to each coating deposition, the chamber was scrubbed with detergent, rinsed with propan-2-ol and acetone (+99%, Fisher Scientific Ltd.), and oven dried. Next, continuous wave air plasma was ignited for 30 min at 0.2 mbar pressure and 50 W power in order to remove any remaining trace contaminants from the chamber walls. Ambient temperature deposition was carried out using a 30 W continuous wave plasma in conjunction with atomization of the precursor into the reaction chamber employing an optimized flow rate of 15 ± 1 × 10^–4^ mL s^–1^. Upon plasma extinction, the atomizer was switched off and the system was evacuated to base pressure, followed by venting to atmosphere.

### 2.2 Refractive Index

The refractive indices and thicknesses of coated silicon wafer substrates were measured using a spectrophotometer (model nkd-6000, Aquila Instruments Ltd.). The obtained transmittance–reflectance curves (a 350–1000 nm wavelength range and a parallel (*p*) polarized light source at a 30° incident angle) were fitted to the Cauchy model for dielectric materials, using a modified Levenberg–Marquardt method (version 2.2 software, Pro-Optix, Aquila Instruments Ltd.).^47,48^

### 2.3 UV–Vis Transmittance Spectroscopy

UV–vis transmittance spectra of coated quartz slides were acquired in the 200–1000 nm wavelength range using a UV–vis–NIR spectrophotometer (model Cary 5000 UV–vis–NIR, Agilent Technologies Inc.).

### 2.4 Infrared Spectroscopy

Fourier transform infrared (FTIR) analysis was carried out using an FTIR spectrometer (model Spectrum One, Perkin Elmer Inc.) equipped with a liquid nitrogen-cooled mercury–cadmium–telluride (MCT) detector. The spectra were averaged over 100 scans at a resolution of 4 cm^–1^ across the 450–4000 cm^–1^ wavenumber range. Attenuated total reflection (ATR) spectra of 4-bromostyrene, 9-vinylcarbazole, toluene, and trimethoxyoctylsilane-TiO_2_ nanoparticles were obtained using a single-reflection diamond brazed into a tungsten carbide accessory (model Golden Gate, Specac Ltd.). Reflection-absorption infrared spectroscopy (RAIRS) of ASPD hybrid and nanocomposite layer-coated silicon wafers was performed using a variable angle reflection-absorption accessory (Specac Ltd.) fitted with a KRS-5 polarizer (to remove the s-polarized component) set at an angle of 55° to the substrate normal (sampling depth of 0.5–20 μm for RAIRS^49^).

### 2.5 Scanning Electron Microscopy

ASPD-coated silicon wafer substrates were mounted onto carbon disks supported on aluminum stubs and then coated with a thin evaporated gold layer (5–10 nm, Polaron SEM Coating Unit, Quorum Technologies Ltd.). Electron micrographs were acquired using a scanning electron microscope (model Vega 3LMU, Tescan Orsay Holdings a.s.) operating in a secondary electron detection mode, in conjunction with an 8 kV accelerating voltage, and a working distance of 12–15 mm. SEM cross-section analysis was performed to cross-check the film thicknesses measured using the spectrophotometer, Supporting Information (Tables S1 and S2). For SEM cross-section analysis, coated silicon wafer substrates were frozen in liquid nitrogen, fractured, and then mounted onto carbon disks supported on 45° tilt aluminum stubs.

### 2.6 Hardness

Hardness and contact modulus measurements were made at a 200 nm depth for ASPD coatings deposited onto silicon wafers using a Hysitron Triboindenter fitted with a general purpose Berkovich diamond tip (tip end radius of 500 nm). Tests were performed under load control to reduce time-dependent deformation effects and were analyzed by the method of Oliver and Pharr.^50^ Twenty-five indentations were made on each coating at a range of loads from 0.1 to 2 mN giving contact depths from 100 to 900 nm. There was some variation of mechanical properties with contact depth, which is due to a combination of time-dependent deformation (which mainly affects hardness) and the presence of the substrate (which mainly affects contact modulus). It is often suggested that the hardness of a coating independent of the substrate can be assessed if the contact depth is less than 10% of the coating thickness and that the elastic modulus of the substrate will contribute to the measurement at a much lower percentage;^51^ the results are reported at a fixed contact depth of 200 nm to minimize this effect.

## 3 Results

### 3.1 Coating Deposition

Photographs of the 4-bromostyrene and 4-bromostyrene/toluene + 8% TiO_2_ ASPD coatings deposited onto silicon wafer, quartz window, and PET polymer film substrates illustrate the substrate-independent nature of the ASPD technique, Supporting Information (Figure S1). Hardness and contact modulus values were measured to be 1.06 ± 0.16 and 40.6 ± 19.9 GPa, respectively, for the 4-bromostyrene ASPD coatings and 0.59 ± 0.18 and 23.0 ± 13.5 GPa, respectively, for the 4-bromostyrene/toluene + 8% TiO_2_ ASPD coatings. Scanning electron microscopy images of the ASPD layers showed that the 4-bromostyrene and 3:2 v/v 4-bromostyrene/toluene surfaces were relatively smooth in appearance, Supporting Information (Figure S2). The incorporation of 9-vinylcarbazole or titania particles into the respective coatings slightly increased the coating surface roughness, with the submicrometer features for the latter case being attributed to the incorporated TiO_2_ nanoparticles, Supporting Information (Figure S2). Spectrophotometry and scanning electron microscopy thickness measurements showed that the atomized spray plasma deposition (ASPD) coatings were uniform in thickness, Supporting Information (Tables S1 and S2). For the 4-bromostyrene precursor, the optimal ASPD film growth rate (325 ± 63 nm min^–1^) was measured to be an order of magnitude greater than that reported for the conventional vapor phase plasma deposition of styrene (10–20 nm min^–1^)^52^ and can be attributed to the higher precursor flow rate associated with the atomization of liquid droplets during ASPD.^37^

### 3.2 Refractive Index

**Figure 2:**
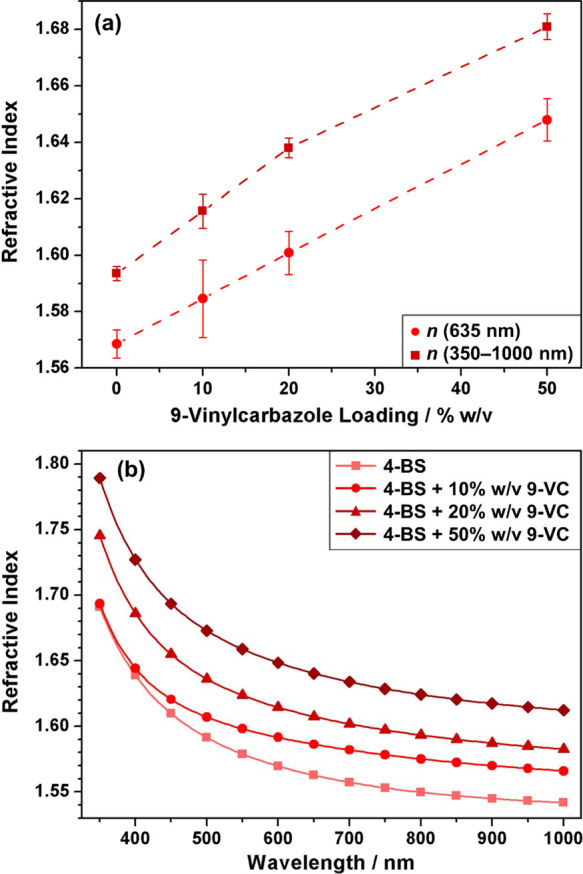
(a) Refractive index at 635 nm and averaged over a 350–1000 nm wavelength range for ASPD 4-bromostyrene–9-vinylcarbazole hybrid layers as a function of 9-vinylcarbazole concentration in the precursor mixture and (b) refractive index variation of ASPD 4-bromostyrene layer and ASPD 4-bromostyrene–9-vinylcarbazole hybrid layers across the 350–1000 nm wavelength range (lines represent different precursor compositions; actual measurements were made every 5 nm between 350 and 1000 nm—a symbol plotted every 50 nm is used to differentiate between the lines). 4-Bromostyrene and 9-vinylcarbazole have been abbreviated to 4-BS and 9-VC, respectively.

The refractive index of the ASPD 4-bromostyrene layer (*n*_635 nm_ = 1.569 ± 0.005) was found to be comparable to the literature value for the 4-bromostyrene precursor (*n*_589.3 nm_ = 1.595^40^), Figure 2. Incorporation of the high refractive index solid compound 9-vinylcarbazole (*n*_poly(vinylcarbazole)_ = 1.683^41^) into the ASPD 4-bromostyrene layer led to an enhancement in the optical properties yielding refractive indices as high as *n*_635 nm_ = 1.648 ± 0.008 for a loading of 50% w/v 9-vinylcarbazole, Figure 2. This improvement in refractive index was across the entire measured wavelength range. For 9-vinylcarbazole concentrations exceeding 50% w/v, the precursor mixture became too viscous to sustain homogeneous atomization. The observed rise in the refractive index value with increasing 9-vinylcarbazole content demonstrates how the optical properties of the ASPD 4-bromostyrene coatings can be easily tuned in order to achieve a desired refractive index by simply varying the 9-vinylcarbazole loading in the precursor mixture.

**Figure 3:**
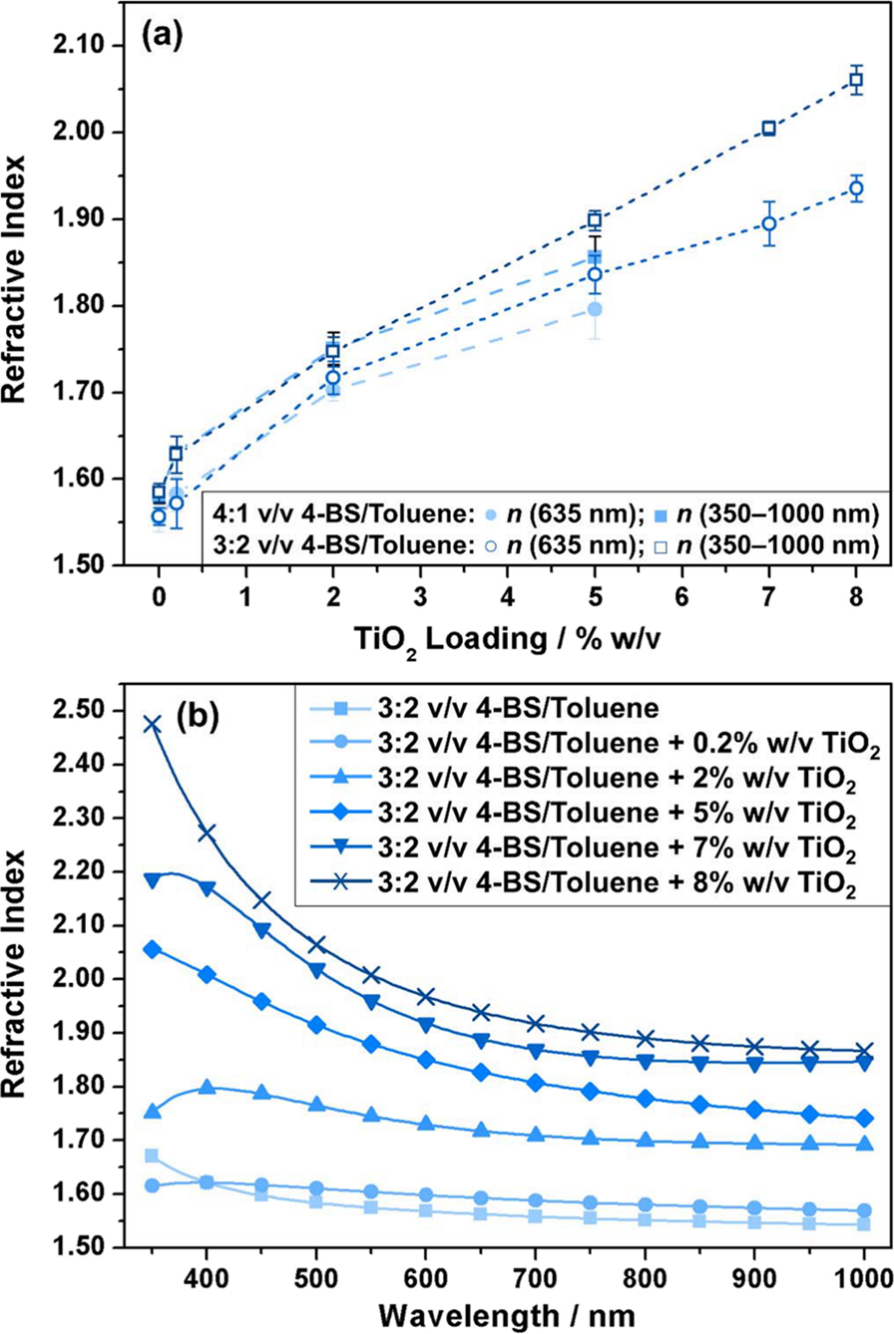
(a) Refractive index at 635 nm and averaged over the 350–1000 nm wavelength range for ASPD 4-bromostyrene/toluene + titania nanocomposite layers as a function of trimethoxyoctylsilane-functionalized titania loading concentration and (b) refractive index variation of the ASPD 3:2 v/v 4-bromostyrene/toluene layer and ASPD 3:2 v/v 4-bromostyrene/toluene + *x*% w/v trimethoxyoctylsilane-TiO_2_ nanocomposite layers across the 350–1000 nm wavelength range (lines represent different precursor compositions; actual measurements were made every 5 nm between 350 and 1000 nm, a symbol plotted every 50 nm is used to differentiate between the lines). 4-Bromostyrene has been abbreviated to 4-BS.

An even greater enhancement in refractive index values was achieved for the case of ASPD 4-bromostyrene–titania nanocomposite layers, Figure 3. This entailed the addition of some toluene to the 4-bromostyrene carrier in order to help disperse the hydrophobic trimethoxyoctylsilane-TiO_2_ nanoparticles within the precursor mixture. In the absence of nanoparticles, the ASPD 4-bromostyrene/toluene (4:1 v/v) layer displayed a slightly lower refractive index (*n*_635 nm_ = 1.555 ± 0.015) compared to the pure 4-bromostyrene coating (*n*_635 nm_ = 1.569 ± 0.005), which is expected due to the addition of a lower refractive index liquid into the precursor mixture (*n*_632.8 nm (toluene)_ = 1.4939).^53^ Incorporation of trimethoxyoctylsilane-TiO_2_ nanoparticles into this host 4-bromostyrene/toluene layer led to a significant increase in refractive index yielding values as high as *n*_635 nm_ = 1.796 ± 0.034 for a precursor slurry loading of 5% w/v titania nanoparticles dispersed in a 4:1 v/v 4-bromostyrene/toluene mixture, Figure 3. The refractive index of these ASPD 4-bromostyrene/toluene + titania nanocomposite layers is greatly enhanced across the entire measured wavelength range (350–1000 nm). The slight drop in refractive index toward lower wavelengths has also been previously observed for other systems, for example, wet-coated polymer–titania composite films, where it is attributed to the incident photon frequency becoming approximately equal to the plasma frequency leading to anomalous dispersion.^54^ For a fixed nanoparticle loading, the refractive index could be increased further by raising the toluene content (*n*_635 nm_ = 1.836 ± 0.022 for 5% w/v titania nanoparticles dispersed in a 3:2 v/v 4-bromostyrene/toluene mixture)—this can be attributed to a better dispersion of nanoparticles in the precursor mixture by using larger amounts of toluene. Control experiments with the titania loading kept constant at 5% w/v while lowering the 4-bromostyrene/toluene ratio further did not lead to any additional improvement in the refractive index beyond this optimal 3:2 v/v value (for instance, 5% w/v TiO_2_ nanoparticles in a 2:3 v/v 4-bromostyrene/toluene mixture gave *n*_635 nm_ = 1.819 ± 0.015). Using this optimal 3:2 v/v ratio of 4-bromostyrene/toluene, the TiO_2_ nanoparticle loading could be extended to beyond 5% w/v. A precursor slurry loading of 8% w/v titania gave refractive indices as high as *n*_635 nm_ = 1.936 ± 0.015, Figure 3. For TiO_2_ nanoparticle concentrations exceeding 8% w/v nanoparticle loading, the precursor mixture became too viscous to sustain homogeneous atomization. This significant enhancement in refractive index values attained by incorporating trimethoxyoctylsilane-TiO_2_ nanoparticles at still relatively low concentrations further demonstrates the capability to fine tune the nanocomposite layer optical properties.

### 3.3 UV–Vis Transmittance Spectroscopy

**Figure 4:**
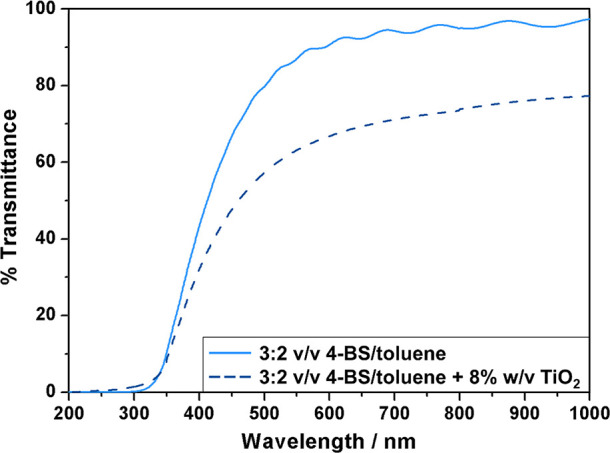
UV–vis transmittance spectra for ASPD 3:2 v/v 4-bromostyrene/toluene layer (solid line) and ASPD 3:2 v/v 4-bromostyrene/toluene + 8% w/v TiO_2_ nanocomposite layer (dashed line). 4-Bromostyrene has been abbreviated to 4-BS.

UV–vis transmittance spectra of the ASPD 3:2 v/v 4-bromostyrene/toluene layers show that the coatings exhibit good transparency in the 450–1000 nm wavelength range, Figure 4. Upon incorporation of 8% w/v trimethoxyoctylsilane-TiO_2_ nanoparticles, the UV–vis transmittance dropped but still exceeded 50% between 450 and 1000 nm. This drop in optical transmittance is most likely due to scattering of the light by the TiO_2_ nanoparticles.^55^

### 3.4 Infrared Spectroscopy

**Figure 5:**
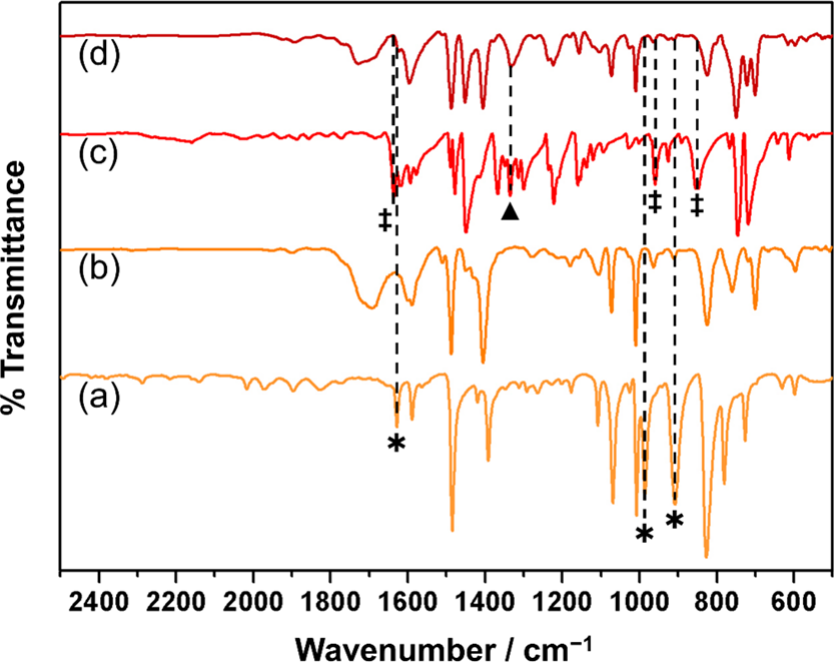
Infrared spectra: (a) the ATR 4-bromostyrene liquid precursor, (b) RAIRS ASPD 4-bromostyrene layer, (c) ATR 9-vinylcarbazole solid precursor, and (d) the RAIRS ASPD 4-bromostyrene–9-vinylcarbazole hybrid polymer layer (50% w/v 9-vinylcarbazole). The asterisk (*) and double dagger (**‡**) denote absorbances associated with the polymerizable vinyl C=C double bond contained in the 4-bromostyrene and 9-vinylcarbazole precursors, respectively. A triangle (▲) denotes C–N stretching absorbance at 1333 cm^–1^. Infrared spectra across the 500–4000 cm^–1^ range are given in the Supporting Information, Figure S3. Assignments are given in the Supporting Information, Table S3.

Infrared spectroscopy showed high levels of structural retention for the ASPD poly(4-bromostyrene) layers, Figure 5. Characteristic poly(4-bromostyrene) ring absorbances include aromatic C–H stretching (3031 cm^–1^ and 3060 cm^–1^), *para*-substituted benzene ring C=C stretching (1488 cm^–1^ and 1590 cm^–1^), and aromatic C–Br (*para*) stretching (1073 cm^–1^).^56,57^ Disappearance of the peaks due to the vinyl C=C bond associated with the precursor molecule confirms polymerization via the 4-bromostyrene vinyl group during ASPD: =CH_2_ wagging (908 cm^–1^), =CH_2_ twisting (986 cm^–1^), and C=C stretching (1629 cm^–1^).^55,58^

The infrared spectrum of the ASPD 4-bromostyrene–50% w/v 9-vinylcarbazole layer clearly shows incorporation of 9-vinylcarbazole into the hybrid polymer layer due to the appearance of an absorption band at 1333 cm^–1^ associated with C—N stretching, Figure 5.^59^ A characteristic *ortho*-substituted benzene ring absorbance is also now observed at 1452 cm^–1^ attributed to the extended 9-vinylcarbazole aromatic structure.^60^ As with the ASPD 4-bromostyrene layer, disappearance of the absorbances associated with the vinyl C=C bond present in the 9-vinylcarbazole precursor is consistent with conventional polymerization taking place at the vinyl C=C double bond: =CH_2_ wagging (854 cm^–1^), =CH_2_ twisting (960 cm^–1^), and C=C stretching (1636 cm^–1^).^56,58^

**Figure 6:**
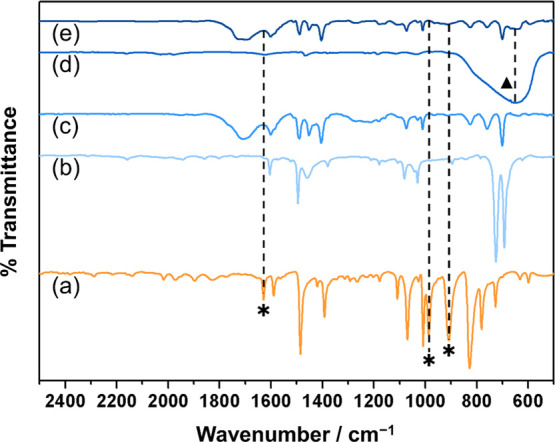
Infrared spectra: (a) ATR 4-bromostyrene liquid precursor, (b) ATR toluene liquid precursor, (c) RAIRS ASPD 3:2 v/v 4-bromostyrene/toluene layer, (d) ATR trimethoxyoctylsilane-TiO_2_ nanoparticles, and (e) RAIRS ASPD 3:2 v/v 4-bromostyrene/toluene + 8% w/v trimethoxyoctylsilane-TiO_2_ nanocomposite layer. An asterisk (*) denotes absorbances associated with the polymerizable vinyl C=C double bond contained in the 4-bromostyrene precursor. A triangle (▲) denotes Ti–O–Ti stretching absorbance at 643 cm^–1^. Infrared spectra the across 500–4000 cm^–1^ range are given in the Supporting Information, Figure S4. Assignments are given in the Supporting Information, Table S4.

For the ASPD 4-bromostyrene/toluene + titania nanocomposite layers, TiO_2_ nanoparticle incorporation throughout the bulk of the layers is evident from the broad absorption band at around 643 cm^–1^ associated with Ti–O–Ti stretching,^61^Figure 6. Aliphatic C–H stretching absorbances at 2868 and 2929 cm^–1^ are indicative of some toluene molecule–plasma reactions, Supporting Information (Figure S4).^55^ This incorporation of toluene into the nanocomposite structure also shifts the characteristic *para*-substituted benzene ring C=C stretching absorbances of 4-bromostyrene toward higher wavenumbers (1489 and 1601 cm^–1^), which is consistent with forming a mixture of *para*- (*para* C=C stretching of 4-bromostyrene precursor: 1485 and 1589 cm^–1^) and mono- (mono C=C stretching of toluene precursor: 1495 and 1604 cm^–1^) substituted benzene rings.^56^

## 4 Discussion

Refractive index is a measure of how fast light travels through a material relative to the speed of light in a vacuum. When light passes through the boundary between two different materials, it changes direction (refraction) due to a change in speed caused by a disruption of electron density at the interface. As a consequence, materials with high polarizability have a high refractive index. Molar refractivity is a measure of the polarizability of a material. For a polymer, the refractive index is dependent upon the molar refractions and molar volumes of both the polymer backbone and the functional groups present.^55^ It can be predicted by summing the molar refractions of all the polymer substituents, and therefore high refractive index polymers contain substituents with a high molar refraction.^62^ Aromatic rings and halogen atoms (except for fluorine) have a high molar refraction due to their large polarizability and high electron density.^10^ Hence, incorporation of these groups into a polymeric coating is an effective way for enhancing its index of refraction. This accounts for the high refractive index value measured for the ASPD 4-bromostyrene layer (*n*_635 nm_ = 1.569 ± 0.005 compared to *n*_589.3 nm_ = 1.595 for the 4-bromostyrene precursor^40^), Figure 2. Infrared spectroscopy of this layer confirms the presence of aromatic groups (aromatic C–H and C=C stretches) and bromine atoms (aromatic C–Br stretch), Figure 5.

Further enhancement in refractive index has been achieved through the mixing of the highly aromatic molecule 9-vinylcarbazole with the 4-bromostyrene precursor reaching refractive indices as high as *n*_635 nm_ = 1.648 ± 0.008 for a loading of 50% w/v 9-vinylcarbazole, Figure 2. An increase in refractive index is measured across the entire wavelength range due to the incorporation of additional aromatic groups (*n*_poly(vinylcarbazole)_ = 1.683^41^). The observed enhancement in refractive index (Δ*n* = +0.079) at maximum 9-vinylcarbazole loading (50% w/v) is in line with previous reports that utilize 9-vinylcarbazole to increase refractive index (*n*) using a blade coating^63^ or moulding process^64^ followed by UV polymerization. Overall, greater refractive indices are achieved in the present study by utilizing a higher refractive index host polymer. Furthermore, syntheses of these earlier reported polymer systems required multiple additives (a cross-linking agent, UV starter to initiate polymerization, and a stabilizer) and UV irradiation and high-temperature baking steps, none of which are required for the single-step ASPD process.

Higher refractive indices are reported previously by incorporating titania into composite coatings using the sol–gel method^19−25^ or by nanoparticle dispersion into polymers^30−32^ and monomer mixtures.^35^ These rely upon the inherently high refractive index of titania (*n* = 2.45 for anatase; *n* = 2.70 for rutile).^20^ This approach has been extended in the present study by dispersing low loadings of trimethoxyoctylsilane-functionalized titania nanoparticles (consisting of a mixture of anatase and rutile^65^) into the 4-bromostyrene precursor for ASPD leading to values as high as *n*_635 nm_ = 1.796 ± 0.034 for a precursor slurry loading of 5% w/v titania nanoparticles dispersed in a 4:1 v/v 4-bromostyrene/toluene mixture, Figure 3. Alkyl group surface functionalization of the TiO_2_ nanoparticles helps dispersion in the 4-bromostyrene precursor.^55^ Toluene addition to the precursor mixture further assists dispersion of the alkyl group-functionalized titania nanoparticles.^42^ The refractive index obtained for the 3:2 v/v 4-bromostyrene/toluene + 8% w/v titania ASPD nanocomposite coating (*n*_635 nm_ = 1.936) exceeds a previous report where titania was added prior to UV-induced liquid-phase monomer polymerization (*n* = 1.861^35^). The refractive index reported here is also higher than for other nanocomposite coatings where nanoparticles were incorporated into a host polymer containing bromine atoms and aromatic rings in order to obtain high refractive index films.^66,67^ Furthermore, the refractive index attained in the current investigation is larger than earlier values for low loading of polymer–inorganic nanocomposite coatings (≤10 wt % inorganic material): *n* = 1.73 (8 wt % TiO_2_),^54^*n* = 1.81 (8 wt % HfO_2_),^68^ and *n* = 1.833 (10 wt % carbon black).^33^ Synthesis of coatings containing titania where the refractive index was slightly higher than the 1.936 value reported in the present study includes wet preparation by nanoparticle dispersion into polymers^32^ and the sol–gel method.^23−25,54^ However, in all of these cases, the titania loading was greater (16–93.4 wt %) than that of employed here, which often results in higher optical loss and reduces the processability of the organic matrix.^34^ Additionally, these methods involve complex synthetic procedures, long processing times, wet steps, high temperatures, and post-deposition curing steps. In contrast, ASPD is a straightforward single-step, dry, low-temperature technique and therefore provides a much simpler approach to depositing high refractive index polymer–titania nanocomposite coatings. It also offers potential for the fabrication of low-refractive index polymer-based coatings through the incorporation of low-refractive index additives.^69,70^ Furthermore, in contrast to conventional spin-coating processes, which require multiple deposition cycles to build up the layer thickness, the ASPD method allows continuous thickness control combined with fast deposition rates.

## 5 Conclusions

High refractive index hybrid polymer and polymer–titania nanocomposite coatings have been prepared in a single step using atomized spray plasma deposition (ASPD). The fabricated polymer–inorganic nanocomposite coatings have very high refractive indices at low titania loadings compared to previously reported polymer–titania coatings (which typically have loadings >30 wt %). This single-step approach offers a dry, low-temperature method for conformably coating any substrate with a high refractive index coating or a coating with a desired refractive index. The simplicity of this approach makes it a promising route for depositing thin films for optical applications.

## Supporting Information Available

Photographs, scanning electron microscopy images, infrared spectra, refractive indices, and coating thicknesses (PDF)

## Notes

Data created during this research can be accessed at https://collections.durham.ac.uk
