# Supplementary material for: Tunable High Refractive Index Polymer Hybrid and Polymer–Inorganic Nanocomposite Coatings
Source: ACS Appl Mater Interfaces. 2021 Jul 13;13(28):33477–84. doi: 10.1021/acsami.1c07372 (PMC8397253; doi:10.1021/acsami.1c07372)
Supplement: Supplementary file 1 — am1c07372_si_001.pdf [file am1c07372_si_001.pdf]

Tunable High Refractive Index Polymer Hybrid and Polymer–Inorganic Nanocomposite Coatings

(Supporting Information)

Angus W. Ritchie[a], Harrison J. Cox[a], Hassan I. Gonabadi[b], Steve J. Bull[b], and Jas Pal S. Badyal[a]*

[a] Department of Chemistry, Durham University, Durham DH1 3LE, England, UK

[b] School of Engineering, Newcastle University, Newcastle-upon-Tyne NE1 7RU, England, UK

* Corresponding author email: j.p.badyal@durham.ac.uk

1. FIGURES

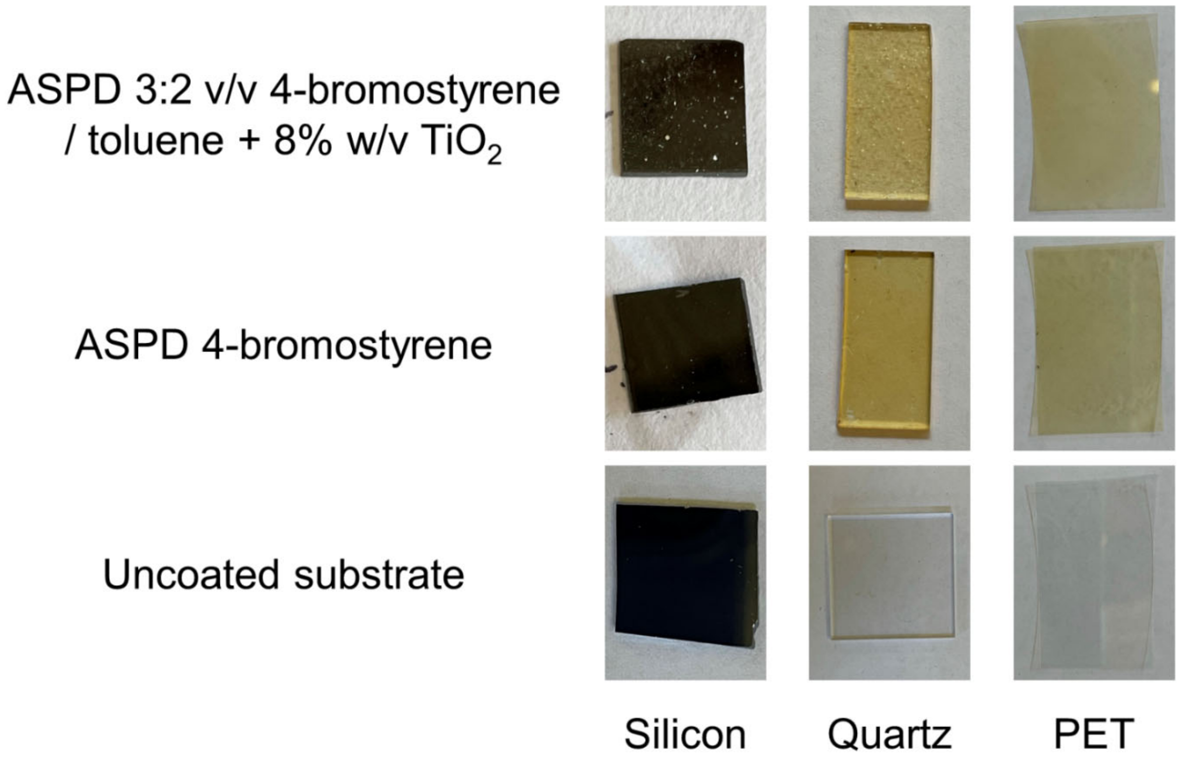

Figure S 1: Photographs of silicon wafer, quartz slide, and PET film substrates for uncoated, ASPD 4-bromostyrene coated, and ASPD 3:2 v/v 4-bromostyrene / toluene + 8% w/v TiO_2 coated.

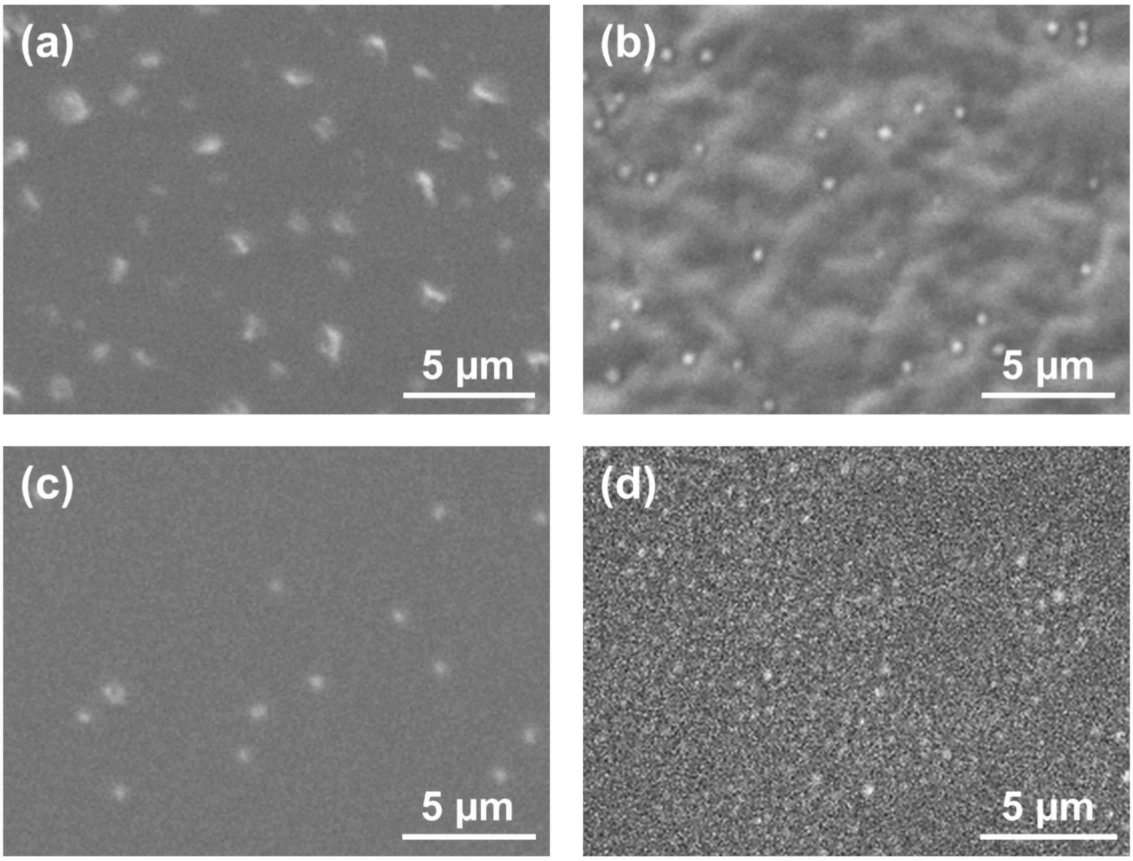

Figure S 2: Scanning electron microscopy (SEM) images of ASPD coatings: (a) 4-bromostyrene; (b) 4-bromostyrene–9-vinylcarbazole (50% w/v 9-vinylcarbazole); (c) 3:2 v/v 4-bromostyrene / toluene; and (d) 3:2 v/v 4-bromostyrene / toluene + 8% w/v TiO_2.

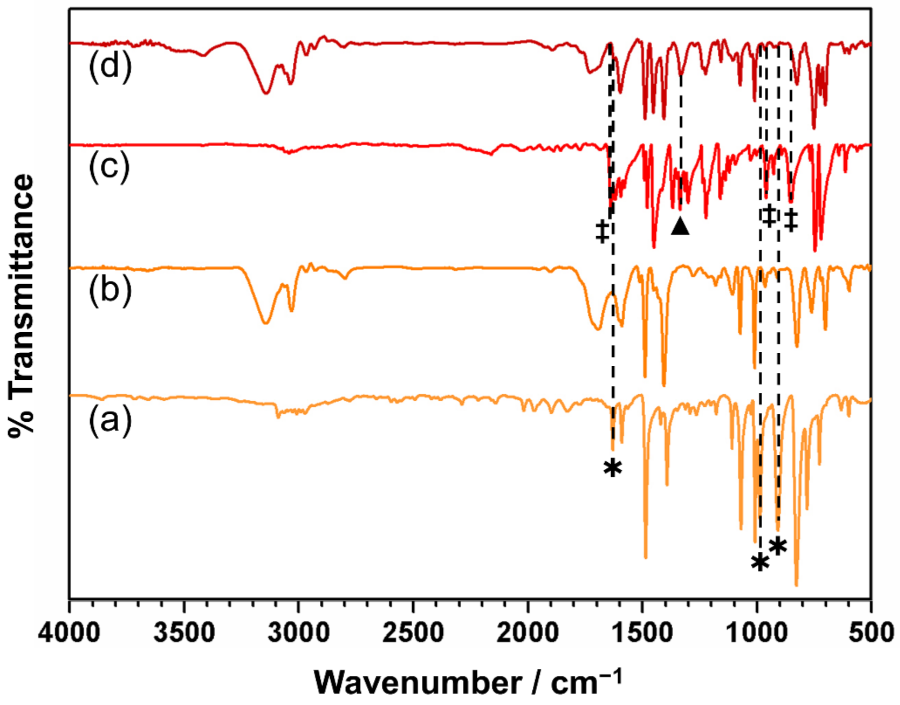

Figure S 3: Infrared spectra: (a) ATR 4-bromostyrene liquid precursor; (b) RAIRS ASPD 4-bromostyrene layer; (c) ATR 9-vinylcarbazole solid precursor; and (d) RAIRS ASPD 4-bromostyrene–9-vinylcarbazole hybrid polymer layer (50% w/v 9-vinylcarbazole). ✱ and ‡ denote absorbances associated with the polymerisable vinyl C=C double bond contained in the 4-bromostyrene and 9-vinylcarbazole precursors respectively. ▲ denotes C–N stretching absorbance at 1333 cm^{-1}. Assignments are given in Supporting Information Table S 3.

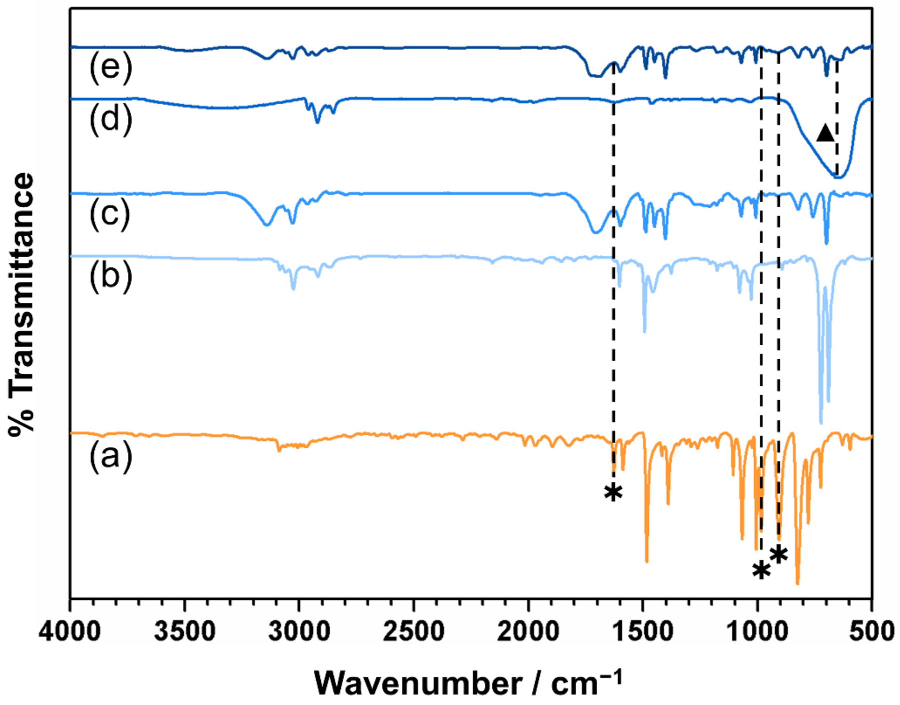

Figure S 4: Infrared spectra: (a) ATR 4-bromostyrene liquid precursor; (b) ATR toluene liquid precursor; (c) RAIRS ASPD 4-bromostyrene / toluene (3:2 v/v) layer; (d) ATR trimethoxyoctylsilane-TiO_2 nanoparticles; and (e) RAIRS ASPD 3:2 v/v 4-bromostyrene / toluene + 8% w/v trimethoxyoctylsilane-TiO_2 nanocomposite layer. ✱ denotes absorbances associated with the polymerisable vinyl C=C double bond contained in the 4-bromostyrene precursor. ▲ denotes Ti–O–Ti stretching absorbance at 643 cm^{-1}. Assignments are given in Supporting Information Table S 4.

2. TABLES

Table S 1: Spectrophotometer refractive index (*n*) (averaged over 350–1000 nm, and at 635 nm), and thickness of ASPD 4-bromostyrene–9-vinylcarbazole hybrid and 4-bromostyrene / toluene + titania nanocomposite layers averaged over multiple coated samples.

**Coating**	**Refractive Index *n***		**Thickness / μm**
	**(350–1000 nm)**	**(635 nm)**	
4-bromostyrene	1.593 ± 0.002	1.569 ± 0.005	1.91 ± 0.32
4-bromostyrene–10% w/v 9-vinylcarbazole	1.616 ± 0.006	1.585 ± 0.014	2.09 ± 0.19
4-bromostyrene–20% w/v 9-vinylcarbazole	1.638 ± 0.004	1.601 ± 0.008	1.81 ± 0.33
4-bromostyrene–50% w/v 9-vinylcarbazole	1.681 ± 0.005	1.648 ± 0.008	2.41 ± 0.72
4:1 v/v 4-bromostyrene / toluene	1.581 ± 0.009	1.555 ± 0.015	2.44 ± 0.28
4:1 v/v 4-bromostyrene / toluene + 0.2% w/v TiO_2	1.631 ± 0.006	1.583 ± 0.020	1.97 ± 0.15
4:1 v/v 4-bromostyrene / toluene + 2% w/v TiO_2	1.751 ± 0.019	1.703 ± 0.012	2.34 ± 0.20
4:1 v/v 4-bromostyrene / toluene + 5% w/v TiO_2	1.856 ± 0.024	1.796 ± 0.034	1.90 ± 0.18
3:2 v/v 4-bromostyrene / toluene	1.584 ± 0.010	1.557 ± 0.010	1.78 ± 0.20
3:2 v/v 4-bromostyrene / toluene + 0.2% w/v TiO_2	1.628 ± 0.021	1.572 ± 0.028	2.05 ± 0.22
3:2 v/v 4-bromostyrene / toluene + 2% w/v TiO_2	1.747 ± 0.017	1.717 ± 0.019	2.18 ± 0.40
3:2 v/v 4-bromostyrene / toluene + 5% w/v TiO_2	1.898 ± 0.011	1.836 ± 0.022	2.09 ± 0.10
3:2 v/v 4-bromostyrene / toluene + 7% w/v TiO_2	2.005 ± 0.008	1.895 ± 0.026	2.00 ± 0.06
3:2 v/v 4-bromostyrene / toluene + 8% w/v TiO_2	2.060 ± 0.017	1.936 ± 0.015	2.02 ± 0.16
2:3 v/v 4-bromostyrene / toluene + 5% w/v TiO_2	1.908 ± 0.032	1.819 ± 0.015	2.10 ± 0.11

Table S 2: Comparison of thickness measurements using spectrophotometer and scanning electron microscopy (SEM) of individual ASPD 4-bromostyrene–9-vinylcarbazole hybrid and 4-bromostyrene / toluene + titania nanocomposite coatings.

**Coating**	**Thickness / μm**	
	**Spectrophotometer**	**SEM**
4-bromostyrene	2.00	1.96 ± 0.02
4-bromostyrene–50% w/v 9-vinylcarbazole	3.15	3.18 ± 0.18
3:2 v/v 4-bromostyrene / toluene	2.00	1.89 ± 0.10
3:2 v/v 4-bromostyrene / toluene + 8% w/v TiO_2	1.94	1.90 ± 0.05

Table S 3: Infrared assignments for ASPD 4-bromostyrene–9-vinylcarbazole hybrid layers.

Assignment	Absorption Frequency / cm^{-1}				Ref
	**4-bromostyrene monomer**	**ASPD 4-bromostyrene**	**9-vinylcarbazole solid**	**ASPD 4-bromostyrene–50% 9-vinylcarbazole**	
Aromatic C–H stretching	3027, 3063, 3088	3031, 3060	3025, 3059, 3078, 3085	3036, 3058, 3084	1
Vinyl C–H stretching	2991, 3008, 3046	–	3014, 3042	–	1
Vinyl C=C stretching	1629	–	1636	–	1
Ring C=C stretching	1485, 1589	1488, 1590	1450, 1594	1452, 1488, 1596	1, 2
C–N stretching	–	–	1335	1333	3
C–Br stretching (Aryl–Br)	1069	1073	–	1073	4
C–C stretching (ring breathing)	1008	1010	1002	1010	1
Vinyl $=CH_2$ twisting	986	–	960	–	1
Vinyl $=CH_2$ wagging	908	–	854	–	1
Aromatic CH deformation out-of-plane	781, 827	825	719, 746	750, 825	1

Table S 4: Infrared assignments for ASPD 4-bromostyrene / toluene + titania nanocomposite layers.

Assignment	Absorption Frequency / cm^{-1}					Ref
	4-bromostyrene monomer	Toluene	ASPD 4-bromostyrene / toluene (3:2 v/v)	Trimethoxyoctylsilane-TiO_2 solid	ASPD 4-bromostyrene / toluene (3:2 v/v) + 8% TiO_2	
Aromatic C–H stretching	3027, 3063, 3088	3026, 3061, 3086	3029, 3059, 3086	–	3028, 3059, 3084	1
Vinyl C–H stretching	2991, 3008, 3046	–	–	–	–	1
Aliphatic C–H stretching	–	2870, 2920	2868, 2929	2852, 2878, 2921, 2960	2872, 2928	1
Vinyl C=C stretching	1629	–	–	–	–	1
Ring C=C stretching	1485, 1589	1495, 1604	1489, 1601	–	1488, 1601	1
C–Br stretching (Aryl–Br)	1069	–	1073	–	1073	4
C–C stretching (ring breathing)	1008	–	1010	–	1010	1
Vinyl $=CH_2$ twisting	986	–	–	–	–	1
Vinyl $=CH_2$ wagging	908	–	–	–	–	1
Aromatic CH deformation out-of-plane	781, 827	692	701, 825	–	700, 825	1
Ti–O–Ti stretching	–	–	–	646	643	5

3. REFERENCES

[1] Lin-Vien, D.; Colthup, N. B.; Fateley, W. G.; Grasselli, J. G. The Handbook of Infrared and Raman Characteristic Frequencies of Organic Molecules; Academic Press, Inc.: San Diego, 1991.

[2] Reyna-González, J. M.; Aguilar-Martínez, M.; Bautista-Martínez, J. A.; Rivera, E.; González, I.; Roquero, P. Influence of the Acidity Level on the Electropolymerization of *N*-vinylcarbazole: Electrochemical Study and Characterization of Poly(3,6-*N*-vinylcarbazole). *Polymer* **2006**, *47*, 6664–6672.

[3] Li, Y.; Yang, J.; Xu, J. In Situ IR Spectroscopic Study of Poly(N-vinylcarbazole) Film During Electrochemical Doping. *J. Appl. Polym. Sci.* **1996**, *61*, 2085–2089.

[4] Katritzky, A. R.; Lagowski, J. M. Infrared Absorption of Substituents in Aromatic Systems. Part V. Halogeno-compounds. *J. Chem. Soc.* **1960**, 2421–2422.

[5] Pirson, A.; Mohsine, A.; Marchot, P.; Michaux, B.; Van Cantfort, O.; Pirard, J. P.; Lecloux, A. J. Synthesis of SiO_2-TiO_2 Xerogels by Sol-Gel Process. *J. Sol-Gel Sci. Technol.* **1995**, *4*, 179–185.
